# The Buffalo Medical Journal

**Published:** 1845-09

**Authors:** 


### *The Buffalo Medical Journal.*

This is another new laborer in the field of medicine; and, judging from the matter and appearance of the fourth number, which is the only one we have received, it promises to be a valuable accession to the medical periodical literature of the United States. It is edited by Austin A. Flint, M. D., and is issued in monthly numbers of twenty-four pages each.

